# Hospital Meetings

**Published:** 1906-04-14

**Authors:** 


### HOSPITAL MEETINGS.

### WEST LONDON HOSPITAL.

A large number of governors and friends attended at the West London Hospital on the occasion of the annual meeting on April 3.

The Duke of Abercorn, the President, had promised to take the chair, but was unable to do so, not having sufficiently recovered from his recent indisposition. The Duchess of Abercorn presided in his place. The annual report having been read, Mr. G. F. Marshall, Chairman of the House Committee, made a few comments on the balance sheet, pointing out that the subscriptions for 1905 were slightly below those for 1904—namely, £38—and the donations were less by £733. However, they had received £1,400 more in legacies, so that their total receipts were considerably larger for 1905. With regard to the economy of the hospital, the cost per patient had fallen considerably last year, being only just over 1s. per head per out-patient and £67 9s. 1d. per bed; the cost per bed in 1904 having been £72 1s. 4¾d. The average cost of each in-patient had also fallen from £4 6s. 4½d. to £3 18s. 1d.

The Duchess of Abercorn moved the adoption of the annual report and statement of accounts, and after expressing the Duke's regret at his inability to be present that afternoon, said that he had the interests and welfare of the hospital much at heart, and rejoiced to watch its progress and continued prosperity. Referring to the expenditure she said that there were many and weighty reasons why the expenses of a properly equipped modern hospital should tend to increase. There were constant developments taking place, all combining to swell the necessary expenses of a great hospital. Among other things, the enormous increase in operative measures, all costing money; highly trained nurses, and far more of them than were formerly considered necessary; the scientific investigation of both medicine and surgery; the bacteriological departments; the constant use of $x$-rays and electrical apparatus. All these had within the last few years contributed to increase the cost and to try the resources of the exchequer to well-nigh breaking-point. She wished to call special attention to the munificent donation of Mrs. Pickering, who gave a thousand guineas to endow a bed in perpetuity. The Post-graduate College maintained its successful career. From the number of gentlemen attending the lectures and demonstrations it would appear that the College must be a great boon to those who desired to acquire hospital experience without the inconvenience attached to the study of disease in company with a class of junior students. This year marked the jubilee of the hospital, and she asked them all to make a special effort to promote the success of the dinner that was to be held later in the year. Money was wanted for many purposes; the nurses required better accommodation; the casualty and out-patient rooms were sadly deficient, and both claimed immediate attention.

The resolution was seconded by Mr. G. F. Marshall, and carried.

### THE QUEEN'S JUBILEE HOSPITAL FAREWELL.

### NAME ALTERED TO THE KENSINGTON GENERAL HOSPITAL.

Mr. Hayes Fisher presided at the annual meeting of the Queen's Jubilee Hospital on Saturday, March 31st. There was a good attendance of governors and friends, the new out-patient department, in which the meeting was held, being crowded.

Mr. Everitt, the Chairman of the Board of Management, read the report, which stated that Lord Brassey had consented to become President and Lord Kenmare a Vice-President of the hospital. Two hundred and thirty-three in-patients were treated during the year, and the attendances of out-patients numbered 27,614, plus 2,731 casualties. The average cost per occupied bed was £108 5s. 2½d. The total receipts were £2,720, including a legacy of £1,000. The ordinary expenditure was £2,717, and there was expended on extension of building £2,590, on which account there is now a debt of £715. He felt particularly gratified, he said, at being able to present so favourable a report. So far as their funds permitted and their accommodation allowed, they had during the year brought the hospital up to the highest state of efficiency. They were glad that so many influential people were rallying round them, and hoped before long to be in possession of an institution of which they would all be proud. One item they had to regret—the closing of the children's ward for want of funds. That was very sad and depressing, and he hoped some kind friend would soon send them money enough so that it could be re-opened without delay. As to the building, they must go cautiously. Bricks and mortar would not pay for themselves. They had made ends meet that year, but they had a debt altogether of £2,000, and he appealed for a supreme effort to wipe that off before their next meeting.

The adoption of the report for 1905 having been agreed to, the Chairman said that one result of the adoption of the report was that Dr. Benham, the founder of the hospital, became a life trustee on the recommendation of the Board, he having assigned the deeds of the hospital to the trustees. He wished to express his pleasure at the compliment that had been paid to Dr. Benham. Now that he (the speaker) would be having leisure from his Parliamentary duties, he proposed to devote more of it to that hospital, which had so long been struggling with adversity, and had done so much good. As a commencement he would put himself down for an annual subscription of ten guineas. The results they had achieved showed that the hospital continued to be popular with the working classes, notwithstanding the criticisms that had been passed on it, and every figure in their accounts showed the necessity for such an institution in that neighbourhood. These people would not continue to come in such numbers unless they heard well of the hospital, and were well treated when they did come. As to criticism, last year when he took that chair it might have been said that Vesuvius was in eruption. That was so no longer; the lava had quite cooled, and he could endorse the claim that the management was now quite satisfactory. As to their difference with King Edward's Hospital Fund, the only matter in dispute between them really came to a question of what was the definition of a large hospital. In addition to the admirable work being done by the West London Hospital, and by St. George's, it was generally admitted that there was a real necessity in that neighbourhood for a large and thoroughly up-to-date out-patient department, and also for a certain number of beds. The exact number might be a matter for discussion, but he thought it could not be contended that they had gone too far at present with 23. Personally, he should be quite willing to stop when they had reopened the children's ward and had made their first addition of a floor, to give accommodation for another 45 beds, leaving the further two floors with the 90 extra beds until the hospital authorities of London had agreed that they were necessary. As to the management, he agreed with Mr. Everitt when he said that, so far as their funds would admit and their accommodation allowed, they had reached a high state of efficiency. They were now on the same footing as other hospitals, and he thought they should be grateful to Dr. Benham for having seen his way to let the hospital of which as its founder he was naturally proud and solicitous go out of leading strings and vest the deeds in trustees. Some day he was sure his name would be honourably remembered in connection with that institution, in spite of criticisms that had been levelled at him, not always too benevolent. Well, if the hospital was needed and was well managed it ought to be supported. Their total annual subscriptions were only £181—a sum ridiculously small. But it was demonstrable that a large amount of money from that district went to King Edward's Fund. Fulham headed the list of the League of Mercy with £1,181, and a similar amount was collected in Kensington. Those two sums were about equivalent to the total expenditure of that hospital. He therefore thought they had a claim on that Fund. As a definite object for a special effort he would mention that the maintenance of their children's ward would cost from £400 to £500 a year, and they could then treat 64 children. They wanted a few more men like Lord Brassey, and, in particular, a young and energetic man for treasurer.

Lord Brassey was then elected President and Lord Kenmare and Mr. Hayes Fisher Vice-Presidents.

Mr. Everitt then moved that the name of the hospital be changed to the Kensington General Hospital, there being, he argued, many advantages in having a territorial designation. Mr. Williams questioned the wisdom of the alteration, and Dr. Benham, on being appealed to, said he was neutral in the matter, though, being of a conservative turn of mind, had been inclined to favour sticking to their guns.

Eventually, on being put to the vote, the resolution was carried by 23 to 10.

It was then moved that a scheme for the incorporation of the hospital should be prepared and submitted to a subsequent meeting of the governors. This was agreed to, and the customary votes of thanks brought the proceedings to a close.

### ROYAL EYE HOSPITAL.

The annual meeting of governors was held at this hospital on March 28th.

Mr. Matthew Clark, treasurer, was in the chair. The annual report having been adopted, the Acting Secretary, Mr. Edwin Easton, pointed out that though the receipts were £150 in excess of the expenditure, yet as a legacy of £735 was in invested stock, and had to be placed to capital account, the expenditure was actually £585 more than the cash receipts. The attendances of out-patients were 71,008, as against 61,199 in 1904, which accounted for the increase in expenditure. The cost per head for out-patients was only 1s. 11d.

Mr. Walter Green said he had had experience in the working of many hospitals, but he had never known one where the cost of administration was kept so low. The amount under this heading for 1905 was only £235.

The Chairman remarked that it was one of the principles of the hospital never to get into debt, and they never had done so. He considered that they did a great deal with their money.

Mr. Jackson, representing the Hospital Saturday Fund, asked whether anything had been done to increase the accommodation for out-patients.

The Chairman replied that they were in exactly the same position as they were last year, and that they must abandon the hope of being able to purchase any of the adjoining property.

Mr. Arthur W. Ormerod, F.R.C.S., brought forward a suggestion as to a rearrangement of the existing accommodation, which would provide a little more space, and a resolution was adopted urging the necessity of taking steps to meet the urgent need of further accommodation for out-patients by some such rearrangement, seeing that there was no likelihood of securing additional land.

### ROYAL SEA-BATHING HOSPITAL.

The Hon. John Biddulph presided on the 28th ultimo at the 115th annual meeting of the Royal Sea-Bathing Hospital, Margate, held at the offices, 13 Charing Cross. In moving the adoption of the report and accounts for 1905, he pointed out that the ordinary income and ordinary expenditure had about balanced each other. They had £3,000 on deposit, and a legacy since received would bring that up to £5,000 toward the sum required for the alterations and additions contemplated. The patients' payments amounted to about £3,090, and that was a very valuable addition to their income. The electrical department had proved of great value in a large number of cases. The patients admitted during the year numbered 479, who, with 91 in the hospital on January 1, made a total of 570 in-patients for the year. The average daily number of in-patients was 121, and of out-patients 51. The average cost per occupied bed was £70 9s. 9d., and the average cost per occupied bed per week was £1 7s. 1d. He regretted very much the death of Dr. Harnett, who, after being honorary assistant visiting surgeon since 1901, was appointed honorary visiting surgeon in April last. Dr. Heaton had been appointed to succeed him, and Dr. Sawers as honorary assistant surgeon to succeed Dr. Heaton.

The resolution was seconded by Sir Henry Lennard, who said he was sure those who had followed the affairs of the hospital during the past ten years or so would agree that a great improvement had taken place in the condition of the hospital generally. They hoped now soon to get into a thoroughly good state as to the buildings. The matter was receiving the careful attention of the committee, and they hoped before the year was out to have everything in an up-to-date condition.

Mr. Thornton, one of the honorary visiting surgeons, also testified to the immense improvement that had taken place in recent years, which showed itself in the difference in the health of the patients. Twenty years ago their operating theatre was unsuitable and almost inaccessible; the wards were not sanitary; the beds were many of them of the most primitive description, while the dietary was not worthy of a good hospital. Their results then were in proportion to their lack of proper arrangements. Now, however, they had a beautiful hospital, properly appointed. There was no better place in England for the treatment of a disease like tuberculosis. They had verandahs where patients could lie day and night, and their dietary was fuller than that of any other place he knew of. When the improvements they contemplated were complete he believed that everything would be in a thoroughly satisfactory condition. The report was adopted.

